# Mediastinal Mass as an Initial Presentation of Acute Myeloid Leukemia in a Young Man

**DOI:** 10.7759/cureus.41006

**Published:** 2023-06-26

**Authors:** Yagya Ahlawat, Juliet Meir, Cory Benjamin, Amir Steinberg

**Affiliations:** 1 Internal Medicine, Westchester Medical Center, New York, USA; 2 Hematology and Oncology, Westchester Medical Center, New York, USA

**Keywords:** extramedullary leukemia, chloroma, anterior mediastinal mass, myeloid sarcoma, acute myeloid leukemia (aml)

## Abstract

A 29-year-old male, hemodynamically stable, presented with chest pain radiating to the interscapular region, with no fever, cough, dyspnea, or other constitutional symptoms. He had right cervical lymphadenopathy on physical examination. Investigations revealed a 3.1 cm anterior mediastinal nodular mass, peripheral immature blood cells, and thrombocytopenia. Bone marrow core biopsy findings were consistent with acute myeloid leukemia (AML). The mediastinal mass was resected via robotic-assisted thoracoscopic surgery. Histopathology revealed involvement of the mediastinal adipose tissue with myeloid sarcoma. Molecular testing showed TP53 mutation, signifying a poor prognosis. The patient failed several lines of therapy and expired. This case demonstrates an atypical presentation of AML and emphasizes the criticality of early detection in individuals who do not exhibit the usual symptoms associated with the disease. The presence of immature cell lines in peripheral blood should prompt an investigation to determine bone marrow involvement in an otherwise healthy young adult.

## Introduction

Acute myeloid leukemia (AML), although rare overall, is the second most common leukemia. Based on National Cancer Institute's Surveillance, Epidemiology, and End Results (SEER) data from 2016 to 2020 in the United States, AML has an age-adjusted annual incidence rate of 4.1 per 100,000 individuals and a mortality rate of 2.7 per 100,000 individuals [1]. It is characterized by clonal expansion and impaired differentiation of myeloid progenitors within the bone marrow. Myeloid sarcoma (MS) is a rare form of AML characterized by cancer cells forming a solid mass in an extramedullary site. It can present with or without concurrent bone marrow involvement [2]. As this tumor can occur in various parts of the body, the clinical presentation is variable, posing a diagnostic challenge.

This report describes an unusual AML presentation in a young man whose only initial symptom was chest pain, which was later found to be secondary to an MS of the anterior mediastinum. In this patient, the presence of blasts on the complete blood count prompted a bone marrow core biopsy, which led to the diagnosis of AML.

It is important to be aware of rare clinical presentations of AML as the early diagnosis and initiation of therapy impact prognosis and survival.

## Case presentation

A 29-year-old male with a past medical history of bipolar disorder, gastroesophageal reflux disease, and obesity presented with a two-day history of sore throat and chest discomfort to the emergency room. He had also noticed an enlarged lymph node on the right side of the neck for one day. COVID-19 testing was negative, and he was sent home on a course of cefalexin for a presumed upper respiratory tract infection. Over the next few days, he had worsening chest pain with radiation to the interscapular region, prompting him to present to the emergency room again. He denied fever, chills, weight loss, shortness of breath, fatigue, or night sweats. He was a non-smoker and had no family history of malignancy. On examination, he was afebrile and vital signs were hemodynamically stable. Physical examination was remarkable for right cervical lymphadenopathy.

**Figure 1:**
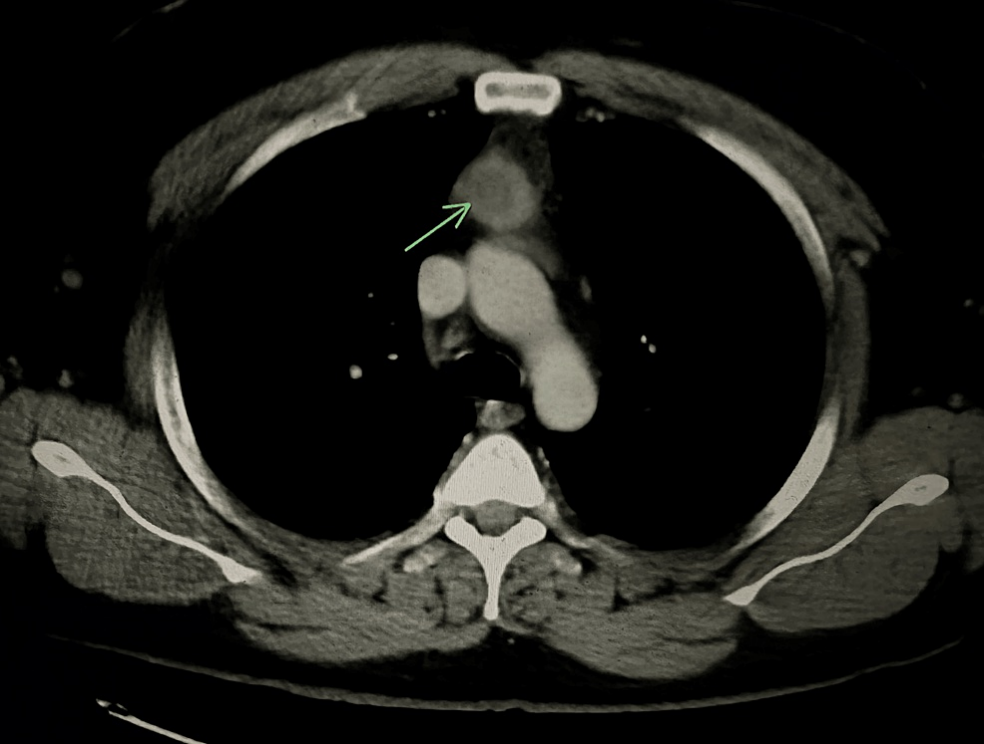
Axial CT image of the chest showing the anterior mediastinal mass CT: computed tomography

**Figure 2:**
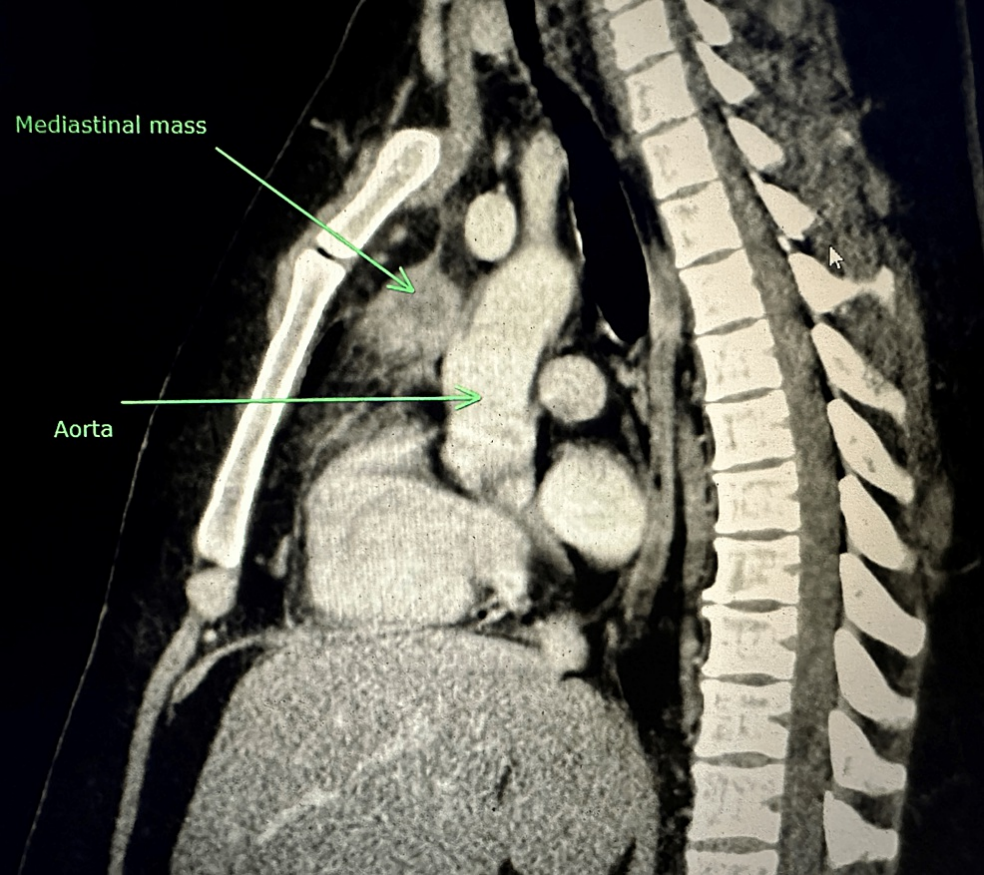
Relation of the mediastinal mass with the aorta in the sagittal view

The laboratory results were notable for a white blood cell count of 11.05 k/mm^3^ with abnormal differential (10% blasts, 1% metamyelocytes, 2% myelocytes, and 6% atypical lymphocytes), hemoglobin of 12.9 g/dL, and a platelet count of 63 K/mm^3^. A computed tomography (CT) scan of the chest with intravenous contrast showed a 3.1 cm anterior mediastinal nodular mass (Figures 1, 2).

Given the patient’s young age at presentation and the presence of a mediastinal mass, an ultrasound of the scrotum was done to evaluate for a germ cell tumor, which demonstrated a left varicocele. Immature cells in the peripheral blood and thrombocytopenia were concerning for bone marrow involvement. 

The bone marrow core biopsy revealed AML. Flow cytometry of the dim CD45 gate with low side scatter revealed an abnormal immature myeloid population (~66% of the total) expressing CD13 (dim), CD33, CD117 (dim), human leukocyte antigen (HLA)-DR, CD11b (subset), CD11c (subset), CD64 (dim), CD71 (dim), CD34 (dim), and myeloperoxidase (MPO) with aberrant expressions of CD4, CD56, and CD9 (dim). These findings were consistent with AML (non-M3 phenotype).

The finding of mediastinal mass or adenopathy is unusual for AML. Given the location of the mediastinal mass being superiorly adjacent to the aorta, there was an additional concern that chemotherapy-induced tumor shrinkage could potentially cause mechanical stress and damage to the blood vessel wall. A robotic-assisted resection of the mediastinal mass via left thoracoscopy was done, along with subtotal thymectomy. Histopathology revealed a thymic mass and mediastinal adipose tissue involved with MS. Immunohistochemical studies revealed atypical cells positive for MPO, CD4, CD117 (weak), and CD34 (weak and partial). Concurrent flow cytometry revealed an abnormal immature myeloid population (~42% of the total) expressing CD13 (dim), CD33, CD117 (dim), HLA-DR, CD64 (dim), CD71 (dim), CD34 (dim), and MPO with aberrant expressions of CD4, CD56, and CD9 (dim).

He received induction chemotherapy using a 7+3 regimen of cytarabine and daunorubicin (seven-day continuous infusion of cytarabine at 100 mg/m^2^/day on days one to seven, and daunorubicin at 90 mg/m^2^/day on days one to three), which aligns with the current standard of care for AML [3]. The course was complicated by pain and hypotension with an unrevealing infectious workup.

Mutational analysis done by next-generation sequencing revealed TP53, RUNX1, and SOCS1 mutations. No mutations were found in the FLT3 and NPM1 genes. Fluorescence in situ hybridization showed deletion in the KMT2A (MLL) gene. Karyotype abnormalities included chromosome 3q rearrangement, loss of chromosome 16, 9-16 translocation, and deletion of 11q. The presence of TP53 mutation and complex karyotype abnormalities conferred poor prognosis, so the role of allogeneic hematopoietic stem cell transplantation (HSCT) following morphologic remission with chemotherapy was discussed with the patient.

Bone marrow core biopsy on day 14 did not show any leukemic cells, but a repeat biopsy on day 21 showed 81% blasts on flow cytometry. The patient was ultimately transferred to an outside hospital for his primary refractory AML but failed several lines of therapy and expired.

## Discussion

AML comprises 1% of all new cancer cases in the US. Although it has been reported in all ages, it is primarily seen in older adults with a mean age of 68 at the time of diagnosis. Approximately one in 10 newly diagnosed cases are persons younger than 35 years of age [1].

The clinical presentation of AML is varied, with many symptoms attributed to pancytopenia. Pancytopenia presents as a result of the excessive proliferation of leukemic cells within the bone marrow, causing a decrease in the synthesis of normal cell lines. The decreased synthesis of red blood cells leads to anemia, which can present with weakness and pallor. Thrombocytopenia can present with signs and symptoms of hemorrhage, petechiae, purpura, epistaxis, and gum bleeding. In addition, a decrease in granulocytes due to the failure of differentiation of immature precursors predisposes patients to infections. Constitutional symptoms, such as weight loss, night sweats, and fatigue, can also be seen.

The above-mentioned patient had symptoms of chest pain and was found to have an anterior mediastinal mass on the CT scan with a biopsy proving MS. Given the location of the mass, the initial differential diagnosis included germ cell tumor, thymoma, and lymphoma [4]. Beta-human chorionic gonadotropin (HCG) and alpha-fetoprotein (AFP) tumor markers were negative, decreasing the likelihood of testicular germ cell tumors [5]. Mediastinal involvement by lymphoproliferative disorders is uncommon, with nodular sclerosing Hodgkin lymphoma and large B-cell lymphoma being the usual culprits [6,7].

MS is a collection of myeloid blasts forming a tumor mass outside the bone marrow and blood. The World Health Organization describes it as a unique presentation of any AML subtype [8]. Commonly affected sites include the bones, lymph nodes, the soft tissues of the head and neck, central nervous system (CNS), and skin [9,10]. It can present concomitantly with bone marrow disease, precede it, or may be seen with AML relapse. It is a rare presentation of AML, seen in about 1-2% of cases, and associated with poor prognosis and short overall survival [11].

Given the rarity and unfavorable outcome associated with this disease, early and accurate diagnosis is important. The above patient was very unusual in his presentation for AML for two primary reasons: younger age (less than 10% of AML cases affect people under 35 years of age) and extramedullary disease (EMD) (seen in about 1% of AML patients) involving mediastinum (EMD more commonly seen in the bones, lymph nodes, and soft tissues; less commonly in the mediastinum).

MS with concurrent AML is treated with intensive AML chemotherapy. Induction therapy commonly involves a combination of cytarabine and an anthracycline. In younger patients with unfavorable-risk AML, HSCT is recognized as the most effective strategy to minimize the risk of disease relapse after achieving remission. For patients with favorable-risk AML and a low risk of relapse, high-dose cytarabine is usually given during the consolidation phase [3]. Managing primary refractory AML poses significant challenges and requires considering factors, such as the patient's age, performance status, and mutational testing results. If a patient is deemed to be a candidate for HSCT, then further chemotherapy regimens are employed to reduce the burden of leukemia cells as a bridge to transplant [12].

## Conclusions

AML typically presents with symptoms related to low blood cell count, such as fever, fatigue, easy bruising, and recurrent infections. MS is a rare presentation of the disease and is associated with a poor prognosis. As the tumor mass can involve any extramedullary site, the clinical presentation is heterogeneous. Given the rarity and unfavorable outcome associated with this, early and accurate diagnosis is important. Moreover, the identification of immature cell lines in peripheral blood should raise suspicion of potential bone marrow involvement in an otherwise healthy young adult, warranting further investigation.
